# Proposing new indicators for glaucoma healthcare service

**DOI:** 10.1186/s40662-017-0071-0

**Published:** 2017-03-07

**Authors:** Yuan Bo Liang, Ye Zhang, David C. Musch, Nathan Congdon

**Affiliations:** 1 Clinical and Epidemiological Eye Research Center, The Eye Hospital of Wenzhou Medical University, Wenzhou, China; 2 Centre for Public Health, Queens University, Belfast, UK; 3 Beijing Tongren Eye Center, Beijing Tongren Hospital, Capital Medical University, Beijing, China; 4 Kellogg Eye Center, University of Michigan, Ann Arbor, MI USA; 5 Eye Hospital, School of Optometry and Ophthalmology, Wenzhou Medical College, No. 270, Xue Yuan Xi Road, Wenzhou, Zhejiang 3250027 China

**Keywords:** Glaucoma coverage rate, Glaucoma detection rate, Glaucoma follow-up adherence rate, Healthcare indicator

## Abstract

Glaucoma is the first leading cause of irreversible blindness worldwide with increasing importance in public health. Indicators of glaucoma care quality as well as efficiency would benefit public health assessments, but are lacking. We propose three such indicators. First, the glaucoma coverage rate (GCR), which is the number of people known to have glaucoma divided by the total number of people with glaucoma as estimated from population-based studies multiplied by 100%. Second, the glaucoma detection rate (GDR), which is number of newly diagnosed glaucoma patients in one year divided by the population in a defined area in millions. Third, the glaucoma follow-up adherence rate (GFAR), calculated as the number of patients with glaucoma who visit eye care provider(s) at least once a year over the total number of patients with glaucoma in given eye care provider(s) in a specific period. Regularly tracking and reporting these three indicators may help to improve the healthcare system performance at national or regional levels.

## Background

Assessing healthcare quality and efficiency has become increasingly important. In the past 20 years, substantial improvements have been seen in cataract blindness prevention. Indicators of cataract surgical rate (CSR) and cataract surgery coverage (CSC) played important roles in evaluating and promoting cataract blindness prevention programs [1]. These indicators provide an evidence base for evaluating the output of all sectors: government, non-governmental organizations, and private sectors. As performance indicators, they measure the extent of the effort to control cataract blindness and allow for comparisons between countries and regions. They also indicate the availability, accessibility, and affordability of cataract services. Such indicators are not yet available for glaucoma even though glaucoma is increasingly important in public health.

Glaucoma is the leading cause of irreversible blindness worldwide. A recent meta-analysis by Tham et al. estimated that the global pooled prevalence of glaucoma among persons aged 40–80 years is 3.54% [2]. In 2013, the number of people with glaucoma worldwide was estimated to be 64.3 million and will increase to 76.0 million in 2020, disproportionately affecting people residing in Asia and Africa [2]. Glaucoma accounts for 12.3% of blindness worldwide [3]. According to Quigley et al., bilateral blindness will be present in 5.9 million people with primary open angle glaucoma (POAG) and 5.3 million people with primary angle closure glaucoma (PACG) in 2020 [4]. With the reduction in blindness due to age-related cataract as access to effective treatment increases [5], glaucoma and diabetic retinopathy will become the two major blindness-causing eye diseases [6, 7]. Thus, glaucoma is a considerable public health issue globally.

Glaucoma can be regarded as a group of chronic eye diseases that have as a common end-point a characteristic optic neuropathy, which is determined by both structural changes (optic disk appearance) and functional deficit (measured by visual field change), with or without an increased intraocular pressure (IOP) [8]. Glaucoma usually affects both eyes, although they may be affected to varying degrees. The public health challenge is that if detected and treated properly with currently available ophthalmic treatments such as hypotensive eye drops, laser or surgery, the disease process can be significantly delayed or possibly prevented. Lack of such treatment is particularly a problem for underserved populations. Detection and treatment of glaucoma fall within the purview of eye care providers, so it is important to evaluate the effectiveness of eye care delivery in glaucoma. We suggest the glaucoma coverage rate (GCR), the glaucoma detection rate (GDR), and the glaucoma follow-up adherence rate (GFAR) as new indicators for evaluating glaucoma care.

## Main text

### Glaucoma coverage rate (GCR) and glaucoma detection rate (GDR)

Even though glaucoma-related blindness is largely preventable with early detection and appropriate treatment regimens, many people who have glaucoma are not diagnosed. For example, in India, studies have found that 91% of persons with open-angle glaucoma were unaware, and 20.3% were already blind bilaterally or unilaterally, respectively, due to glaucoma [9]. In China, results of the Handan Eye Study showed that over 90% of participants with primary angle closure (PAC), over half with PACG and more than 95% of POAG cases had not previously been diagnosed or treated, while 65.6% of PACG, and 4.5% for POAG were blind in at least one eye [10, 11]. Even in developed nations, as many as half of those with glaucoma are unaware that they have the disease [12–14]. Reasons include inadequate screening, unavailability or low utilization of eye care services, and lack of awareness due to the absence of symptoms in the early stages of glaucoma.

The GCR could serve as an important index for evaluating glaucoma healthcare. It is calculated by dividing the number of people in the population with known glaucoma by the total number of people with glaucoma as estimated from population-based studies. However, this parameter can only be obtained through conduct of or access to results from well-designed population-based studies. Practically, we would suggest using the number of patients with newly-detected glaucoma in one year in a defined region divided by the number of people in that defined region, which represents the GDR. With increasing improvement in medical care systems in many countries, the number of detected glaucoma cases can be accurately tracked [15]. The GDR and GCR will vary among populations based on public awareness of the disease, the accessibility and capacity of the regional/national eye care system, existence of user fees, willingness to pay and other related factors.

Although population screening for open angle glaucoma has not been found to be cost effective [16, 17], healthcare planners can use the GDR to track the impact of other, more practical methods to increase glaucoma detection, such as community education [18], screening of targeted high risk groups (including relatives of known glaucoma patients) [19] and enhanced clinic-based case finding through training and incentivizing clinicians to carry out the complete examinations needed to detect asymptomatic glaucoma [20]. With access to estimates of GDR across nations and regions, focused attention could be applied to areas with low GDRs and influence those responsible for allocation of healthcare resources to intervene [21]. Access to a well-established cross-hospital medical information system would provide an important resource to track the number of newly-diagnosed cases.

The formula for GCR/GDR would be:

$$ \mathrm{G}\mathrm{C}\mathrm{R}=\frac{\mathrm{Number}\kern0.5em \mathrm{of}\kern0.5em \mathrm{people}\kern0.5em \mathrm{with}\kern0.5em \mathrm{known}\kern0.5em \mathrm{glaucoma}}{\mathrm{Total}\kern0.5em \mathrm{number}\kern0.5em \mathrm{of}\kern0.5em \mathrm{patients}\kern0.5em \mathrm{with}\kern0.5em \mathrm{glaucoma}\kern0.5em \mathrm{as}\kern0.5em \mathrm{estimated}\kern0.5em \mathrm{from}\kern0.5em \mathrm{population}\hbox{-} \mathrm{based}\kern0.5em \mathrm{studies}}\times 100\% $$

$$ \mathrm{G}\mathrm{D}\mathrm{R}=\frac{\mathrm{Number}\kern0.5em \mathrm{of}\kern0.5em \mathrm{people}\kern0.5em \mathrm{with}\kern0.5em \mathrm{newly}\hbox{-} \mathrm{detected}\kern0.5em \mathrm{glaucoma}\kern0.5em \mathrm{in}\kern0.5em \mathrm{one}\kern0.5em \mathrm{year}}{\mathrm{Number}\kern0.5em \mathrm{of}\kern0.5em \mathrm{people}\kern0.5em \mathrm{in}\kern0.5em \mathrm{a}\kern0.5em \mathrm{given}\kern0.5em \mathrm{a}\mathrm{rea}\kern0.5em \left(\mathrm{in}\kern0.5em \mathrm{millions}\right)} $$

### Glaucoma follow-up adherence rate (GFAR)

As glaucoma is a chronic eye disease, and IOP is the only well-proven modifiable risk factor, lifelong ocular hypotensive medical, laser or surgical treatment is indicated to prevent progression in most cases. Even when glaucoma is detected and treated, inadequate response to therapy and/or IOP fluctuation can cause further damage. This creates the important need for regular follow-up by eye care professionals to monitor glaucomatous optic nerve damage and visual field defects, adjusting therapy as needed [22, 23]. According to recommended clinical practice, even patients with suspected glaucoma and modest risk for progression should be seen at least every 12–24 months, whereas patients with diagnosed glaucoma should have a follow-up visit every 3–6 months [24].

Poor adherence with recommended glaucoma follow-up care serves as a major obstacle to proper disease management. Jin et al. reported follow-up rates at 6, 12 and 48 months after 1186 glaucoma operations in Xian, China as 68.5, 62.1 and 48.8%, respectively [25]. Main risk factors for failed follow-up included low annual income, old age, inability to read, long distance from hospital, and poor disease awareness. Liu et al. reported the follow-up rate in cases of PACG in Handan City, China, at 6, 12 and 48 months after trabeculectomy as 41.1, 21.3 and 13.3%, respectively [26]. They also found that poor knowledge about glaucoma, rural residence, and having poor vision were associated with lower follow-up rates [26]. A recent short-term prospective study found that poor adherence to recommended post-trabeculectomy follow-up was associated with lower education, unawareness of the importance of follow-up, lack of an accompanying person, low family annual income, and not requiring removal of scleral flap sutures postoperatively [27]. The problem of sub-optimal adherence with both post-operative care and medical therapy for glaucoma in developed countries is also well-documented [28–30]. Additional reasons for poor follow-up adherence were identified, such as difficulty on the part of the patient or escort to get time off from work for appointments, long waiting times in the clinics, unfamiliarity with treatment requirements, lack of knowledge regarding the permanency of glaucoma-induced vision loss, cost of examination being too high, and legal blindness [31, 32].

Adherence to follow-up is an essential component of effective care for glaucoma. The follow-up adherence rate can be calculated as the number of follow-up visits that take place within a defined period of time divided by the number of expected/planned visits. The latter number varies greatly due to the differing practice patterns of clinicians and the stage of glaucoma. For example, during the early post-operative period, more frequent visits are needed, whereas less frequent visits are needed when a patient’s glaucoma status is stable. Based on a public health perspective, we recommend GFAR, calculated as the number of patients with glaucoma who visit eye care provider(s) at least once in a year’s period divided by the total number of patients with glaucoma diagnosed in given eye care center(s), as another essential index for the evaluation of glaucoma healthcare.

The formula for GFAR would be:

$$ \mathrm{GFAR}=\frac{\begin{array}{l}\mathrm{Number}\kern0.5em \mathrm{of}\kern0.5em \mathrm{glaucoma}\kern0.5em \mathrm{patients}\\ {}\mathrm{with}\kern0.5em \mathrm{a}\mathrm{t}\kern0.5em \mathrm{least}\kern0.5em \mathrm{one}\kern0.5em \mathrm{visit}\kern0.5em \mathrm{a}\kern0.5em \mathrm{year}\end{array}}{\begin{array}{l}\mathrm{Number}\kern0.5em \mathrm{of}\kern0.5em \mathrm{patients}\kern0.5em \mathrm{with}\kern0.5em \mathrm{glaucoma}\\ {}\mathrm{diagnosed}\kern0.5em \mathrm{in}\kern0.5em \mathrm{given}\kern0.5em \mathrm{eye}\kern0.5em \mathrm{care}\kern0.5em \mathrm{center}\left(\mathrm{s}\right)\end{array}}\times 100\% $$

There are some strategies that can be taken within the healthcare system to improve adherence to follow-up in glaucoma patients. Suggested measures include: 1) educating current and in-training eye care providers on proven communication strategies for improving follow-up; 2) reducing or eliminating fees for post-operative examinations and consider incentives such as provision of free medication at postoperative visits, due to the particular importance of good compliance during this period; 3) providing visit reminders (e.g., via text or telephone) or a support network such as a case manager or glaucoma patient club to help patients adhere to the management requirements of their eye condition.

The GDR, GCR, and GFAR, as proposed above, are sometimes very difficult for a country or region to estimate, especially so for those with limited healthcare systems and less accurate data to rely upon. Governments in most countries are responsible for covering at least some portion of eye care costs and investment in low vision rehabilitation and care as well as monitoring and improving the GDR, GCR, and GFAR are likely to reduce healthcare costs in the long run. The limitation of using these indicators is the lack of a threshold value for judging whether these indicators are reflective of good or inadequate detection and care of glaucoma based on limited studies. However, upon measuring these indicators, they can be used for self-comparison or cross regional comparison.

## Conclusion

In conclusion, from a public health perspective, we need standard indices to compare and evaluate the level of glaucoma care across different countries and regions, with the goal to improve the prevention and treatment outcome of glaucoma, which is the leading cause of irreversible blindness. We propose that GDR, GCR, and GFAR may be particularly useful in this respect.

## Abbreviations

CSC: Cataract surgical coverage
CSR: Cataract surgery rate
GCR: Glaucoma coverage rate
GDR: Glaucoma detection rate
GFAR: Glaucoma follow-up adherence rate
IOP: Intraocular pressure
PAC: Primary angle closure
PACG: Primary angle closure glaucoma
POAG: Primary open angle glaucoma
